# High Dose of Nickel Unbalances Carbon Metabolism and Nitrogen Assimilation in Barley (*Hordeum vulgare* L.)

**DOI:** 10.3390/plants14182927

**Published:** 2025-09-20

**Authors:** Alessia De Lillo, Ivana De Rosa, Giorgia Capasso, Giorgia Santini, Concetta Di Napoli, Noemi Russo, Ermenegilda Vitale, Stefania Grillo, Sergio Esposito, Simone Landi

**Affiliations:** 1 Dipartimento Biologia, Università di Napoli “Federico II”, Complesso Universitario Monte Sant’Angelo, Via Cinthia, 80126 Napoli, Italy; 2 National Research Council of Italy, Institute of Biosciences and Bioresources (CNR-IBBR), Research Division Portici, 80055 Portici, Italy

**Keywords:** heavy metals stress, Poaceae, carbon metabolism, nitrogen assimilation, Nickel, crops

## Abstract

Pollution from heavy metals represents one of the most important threats to crops. Among these, Nickel (Ni) represents a dangerous element, strictly related to anthropic activity and easily accumulated in plants. In this study, effects of high levels (1 mM) of Ni^2+^ were investigated in barley (*Hordeum vulgare* L. cv. Nure) grown hydroponically, inducing a severe reduction in plant growth, as well as genotoxic damage. Moreover, stress affects photosynthesis, inducing a decrease in F*v*/F*m* and ΦPSII and an increase in D1 protein and RuBisCO (RbcL) abundance to compensate for the loss of photosynthetic efficiency. Changes were observed in carbon metabolism, with increases in phosphofructokinase, glyceraldehyde-3P dehydrogenase-NAD^+^, and pyruvate kinase expression confirmed by increased proteins and activities. Notably, there was an evident rise in PEP carboxylase activity, presence, and expression. This increase boosts the TCA cycle (increased fumarase) and supports photorespiration. Evident rises were observed also for glucose-6P dehydrogenase activity and presence. Ni^2+^ stress induced an evident increase in enzymes involved in nitrogen metabolism: particularly, the chloroplastic GS2/Fd-GOGAT cycle and N assimilation through the cytosolic glutamate dehydrogenase reaction were enhanced. These results design a specific stress-responsive metabolism by diverting the synthesis of N-compounds through alternative C/N assimilation pathways to counteract the effects of Ni^2+^ toxicity. This study depicts a diversion of the main C/N metabolism network towards an increase in leaf N assimilation, using carbon skeletons from dark CO_2_ fixation under high Ni^2+^ stress. These results may provide possible targets for the improvement of heavy metal tolerance in cereals.

## 1. Introduction

Plant nutrition is based on a specific balance of macro- and micronutrients required for their healthy growth and correct development. These elements, mostly heavy metals (HMs), play different biochemical and physiological roles, acting as key components of many cellular enzymes and participating in essential redox reactions.

Metals are absorbed by specific transporters located on the root surface and, in some cases, through foliar uptake in trace amounts [1]; then, HMs can be transported, distributed, and stored in plant tissues and cells [2].

Although HMs are naturally present in soils, their concentrations have undergone significant changes because of human activities, including intensive cultivation, mining, manufacturing processes, and improper disposal of industrial waste [3]. These anthropogenic inputs have resulted in both deficiencies and accumulations of heavy metals (HMs) in crop fields, raising growing concerns about their impact on ecosystems [4].

Over the years, it has been well established that elevated levels of HMs have detrimental effects on plant cells, modifying their structure and disrupting their physiology [5,6].

Specific heavy metals (HMs), including Nickel (Ni), represent micronutrients essential during the plant life cycle [7]. Nickel, mainly present in soils as a Ni^2+^ cation, is recognized as an essential micronutrient for plants. It plays a critical role in plant metabolism, serving as a structural component for key enzymes such as urease, Ni-superoxide dismutase, and hydrogenase, and functioning as an activator for various other enzymes [8]. On the other hand, Nickel—a transition element—is of increasing concern due to its elevated concentrations in the soils across various regions, including Asia, North America, and Europe. Thus, despite its physiological relevance, Ni^2+^ can become toxic when accumulated beyond threshold levels, as commonly observed for other micronutrients [9].

Soils exhibit a wide range of Ni^2+^ concentrations, typically from 50 to 2000 mg/Kg, depending on both natural conditions and anthropogenic inputs [10]. Its mobility and bioavailability are modulated by soil pH, concentration of cations and anions, particle size, and osmotic potential [11].

Prolonged exposure to high doses of Ni^2+^ is known to induce various toxic effects, including alterations in growth, plant water status, photosynthetic rate, pigment composition, and the onset of oxidative stress [12,13,14]. Ni^2+^ general toxicity thresholds are approximately 10 mg/Kg for sensitive plant species and up to 50 mg/Kg for moderately tolerant species [15].

Conversely, certain hyperaccumulator species—often used for phytoremediation of contaminated soils—are capable of tolerating and accumulating higher concentrations [16]. It should be underlined that, in the same family [17] or even in the same species [18], Ni^2+^ uptake, accumulation, and distribution in different organs can strongly change.

In *Solanum lycopersicum* L., soil Ni^2+^ concentrations around 300 mg/Kg have been associated with osmotic responses aimed at counteracting Ni^2+^ uptake. Similar adaptive mechanisms have been observed in other species, indicating that water regulation acts as a specific strategy to mitigate metal-induced stress and Ni^2+^ toxicity [15].

In contrast, species within the Poaceae family, including all major cereal crops, tend to exhibit Ni^2+^ toxicity at much lower concentrations, typically between 100 and 200 μM in hydroponic systems [19,20].

Important cereal crops sensitive to Ni^2+^ are wheat (*Triticum aestivum* L.) [21], millet (*Pennisetum glaucum*), and oat (*Avena sativa* L.) [20], in which Ni^2+^ affects seedling growth, lipid peroxidation, total chlorophyll, proline content, and enzymatic activities. Studies on maize (*Zea mais* L.) confirmed a strong accumulation of Ni^2+^ in the roots and a consistent translocation to the aerial parts of the plants [9]. Particularly, in rice (*Oryza sativa* L.), exposure to elevated soil Ni^2+^ levels induced a range of physiological disturbances. Notably, Ni^2+^ exposure negatively affects plant water relations by interfering with processes such as osmosis and diffusion. Under Ni^2+^ stress, parameters like leaf water potential, stomatal conductance, transpiration rate, and total moisture content are significantly reduced [19]. On the other hand, it should be underlined that a survey on over 70 diverse varieties of rice exposed to Ni^2+^ resulted in a wide range of susceptibility and different responses [18]. As regards barley (*Hordeum vulgare* L.), the literature data of growth in hydroponic culture at 100 µM of Ni^2+^ report typical visual symptoms of Ni^2+^ toxicity, such as chlorosis, leaf necrosis, and browning of the root system [22]. Nevertheless, despite these well-documented physiological and biochemical responses, knowledge about the specific effects of Ni^2+^ on primary metabolism remains limited [8], particularly considering that barley is the fourth most widely cultivated crop worldwide [FAOSTAT 2023, http://www.fao.org/faostat/, (accessed on 1 July 2025)] and a widely studied model organism for cereal improvement and stress response.

As recently reported, Ni^2+^ stress can severely affect photosynthesis, leading to structural damage to chloroplasts, reduced photosynthetic efficiency, and impaired carbon assimilation [23,24]. Furthermore, the photosynthetic capabilities of plants appear to be highly influenced by Ni^2+^, not only due to the obvious effects on cellular structure, but also because of the uncoupling of electron flow in the chloroplasts.

In addition, key metabolic pathways, such as carbon metabolism (glycolisis, TCA cycle) and nitrogen assimilation (inorganic nitrogen uptake, GS-GOGAT cycle), are particularly vulnerable, resulting in altered enzyme expression and activity levels [25]. In tomato, it has been shown that Ni^2+^ stress increased inorganic nitrogen uptake and activated enzymes of N assimilation; interestingly, these results were confirmed by transcriptomic analysis [25].

These effects differ depending on Ni^2+^ concentration and exposure period; in this context, the distinctive metabolic alterations induced by Ni^2+^ should be characterized. Therefore, a better knowledge of the effects of Ni^2+^ in barley is desirable.

In this study, we aim to advance the understanding of the response of barley plants (*H. vulgare* L. cv. Nure) to Ni^2+^ exposure by investigating potential alterations in main metabolic pathways and enzymes involved in carbon and nitrogen assimilation and utilization.

The findings could provide a theoretical basis for the bioremediation of Ni^2+^-contaminated farmland (e.g., screening high-accumulating cultivars) or precision fertilization strategies (e.g., adjusting nitrogen supply to mitigate toxicity).

Overall, the data presented here seek to contribute to a broader overview of the metabolic changes occurring in higher plants under Ni^2+^ stress, and more generally in response to HM exposure. Particularly, this study is the first to reveal the coordinated regulatory network of carbon and nitrogen metabolism in barley under Ni^2+^ stress at the transcriptomic and metabolic levels, providing potential targets for the genetic improvement of heavy metal tolerance in cereal crops.

## 2. Results

Barley plants were grown in hydroponics, as these conditions exacerbate the effects of HM stress with respect to soil cultivation, both in pots and open fields [11].

Preliminary experiments using different Ni^2+^ levels from 0.5 mM to 2 mM indicated that visible effects in barley plants, such as leaf chlorosis and wilting, were caused by 1 mM Ni^2+^ exposure within the first 24 h. Therefore, we chose to expose plants to 1 mM Ni^2+^, sampling roots and leaves up to 7 d. Due to the well-known cumulative effects of HMs on plants, these conditions would resemble those of a long-term exposure to Ni^2+^ in polluted soils. Ni^2+^ accumulated over 5000 μg Ni^2+^ ∙ g^−1^ DW in the roots, and 130 μg Ni^2+^ ∙ g^−1^ DW in the leaves (Supplemental Table S2).

### 2.1. Effects of Ni^2+^ on Barley Growth

**Figure 1:**
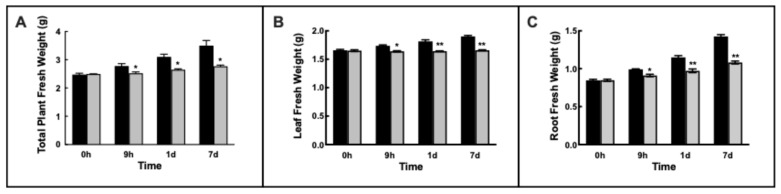
(**A**) Changes in fresh weight of barley plants grown under control condition (black bars) or exposed to 1 mM Ni^2+^ at different times (grey bars); (**B**) changes in fresh weight of barley leaves grown under control condition (black bars) or exposed to 1 mM Ni^2+^ at different times (grey bars); (**C**) changes in fresh weight of barley roots grown under control condition (black bars) or exposed to 1 mM Ni^2+^ at different times (grey bars). Results are average of five to ten different measurements ± standard error (error bars). Asterisks indicate *p* values (* *p* ≤ 0.05, ** *p* ≤ 0.001) in Ni^2+^-exposed plants with respect to control plants at the same time of sampling.

Barley plants showed continuous growth under control conditions: single plant FW increased from 2.5 to 3.3 g in 7 d (Figure 1A). This growth is due both to the leaves (20%—Figure 1B) and roots (70%—Figure 1C).

Ni^2+^ exposure induced a progressive and significant slowdown of the growth: after 7 d growth, the increase observed was 9% (Figure 1A). Specifically, leaf FW remained substantially unchanged (Figure 1B), and root FW was increased by 30%, less than half of that of the control plants (Figure 1C).

Water Content (WC) and Relative Water Content (RWC) in the leaves were measured after 1 mM Ni^2+^ exposure: a minor increase (about 15%) in Water Content was observed in leaves (Supplemental Figure S1A). RWC did not change upon Ni exposure, suggesting that Ni-treated leaves have a slightly lower DW, while leaf hydration was not apparently altered by Ni (Supplemental Figure S1B).

### 2.2. Genotoxic Damage Induced by Ni^2+^ Exposure

Ni^2+^ induced genotoxic damage in barley leaves. Preliminarily, we observed a light DNA damage using RAPD analysis, revealing the presence of different amplification patterns in at least 1 out of 8 primers tested (Supplemental Figure S2).

**Figure 2:**
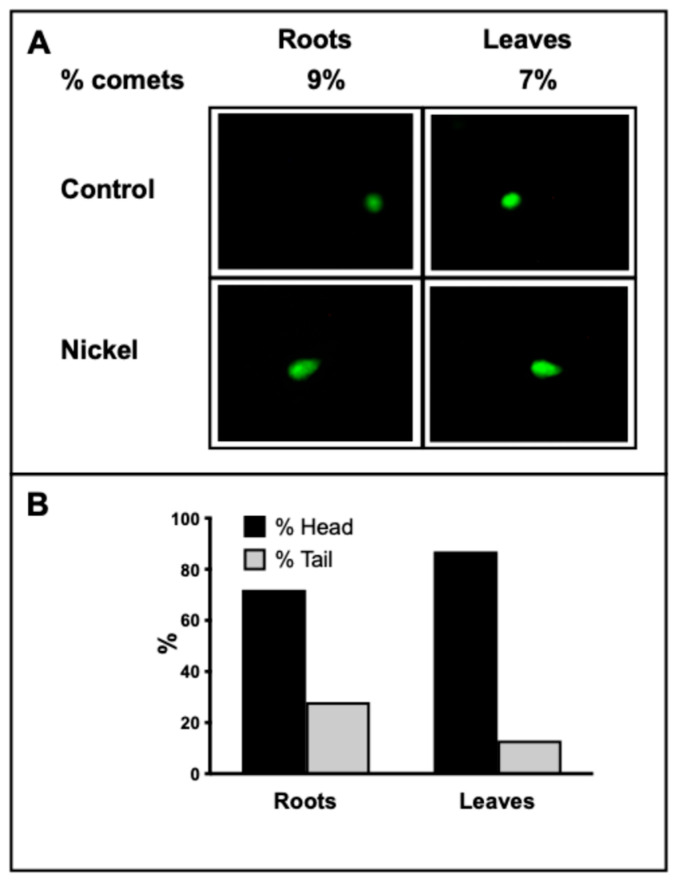
(**A**) Images of the nuclei obtained after Comet assays made on control and plants exposed to 1 mM Ni^2+^ for 7 days. (**B**) Graph showing the ratio between “head” and “tail” observed in roots and leaves, respectively.

Comet assays were made on both leaves and roots extracts from 1 mM Ni^2+^-exposed plants (Figure 2A). Comets obtained revealed that 28% and 13% of DNA could be found in the tails of roots and leaves, respectively. Moreover, 9% of nuclei in the roots and 7% in the leaves showed the typical comet shape, indicating a low—but present—damage after the exposure of plants to 1 mM Ni^2+^ (Figure 2B).

### 2.3. Changes in Main Photosynthetic Parameters

The analysis of the photochemical indexes showed that Ni^2+^ significantly modifies the functionality of the photosynthetic apparatus in barley leaves. Namely, Ni^2+^ effects strongly depended on time-length of exposure to the pollutant. To better follow these effects, we chose to sample leaves also at 2 d and 3 d after Ni^2+^ exposure.

**Figure 3:**
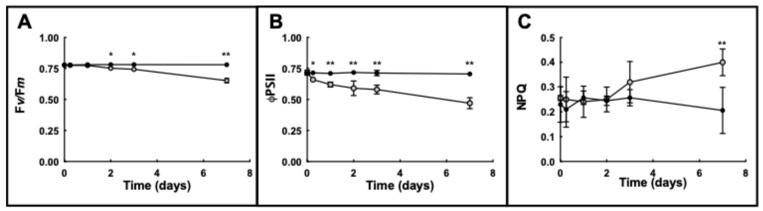
Variations in main photosynthetic parameters in leaves of barley plants grown under control conditions (black circles) or exposed to 1 mM Ni^2+^ (grey circles) at given times. (**A**) Chlorophyll fluorescence parameter F*v*/F*m* ratio; (**B**) quantum yield ΦPSII; (**C**) non-photochemical quenching (NPQ). Results are average of at least three different measurements ± standard error (error bars). Asterisks indicate *p* values (* *p* ≤ 0.05, ** *p* ≤ 0.001) in Ni^2+^-exposed plants with respect to control plants at the same time of sampling.

After two days of exposure to Ni^2+^, barley plants showed a significant and progressive reduction (*p* < 0.01) of the maximum photochemical efficiency of PSII (F*v*/F*m*). In Ni^2+^ leaves, F*v*/F*m* ratio was over 15% lower than controls after 7 d (Figure 3A).

The quantum yield of PSII electron transport (ΦPSII) is a more sensitive index compared to F*v*/F*m*, showing a significant (*p* < 0.01) decline 9 h after Ni^2+^ treatment compared to the untreated control. ΦPSII gradually declined with time, reaching the lowest value after 7 d, when a 33% decline in ΦPSII was observed (Figure 3B).

Concomitantly to the decline of ΦPSII, non-photochemical quenching (NPQ) remained unchanged during the first 2 d of exposure to Ni^2+^. Afterwards, NPQ increased continuously by 50% over control level after 7 d (Figure 3B).

### 2.4. Enzymatic Activities and Changes in Proteins upon Ni^2+^ Exposure

In barley grown under control conditions, PEPCase activity was 44.9 ± 1 nmol min^−1^ mg^−1^ protein in roots; 1 mM Ni^2+^ caused a slight 17% increase in activity up to 53.6 ± 1 nmol min^−1^ mg^−1^ protein after 1d. After 7d, activity returned to the initial value.

**Figure 4:**
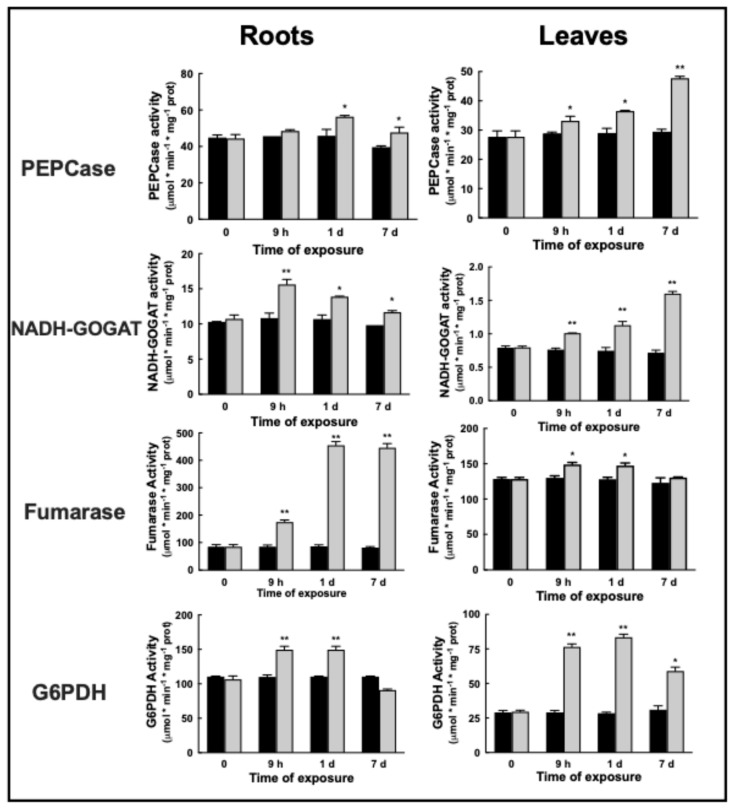
Changes in enzymatic activities of phosphoenolpyruvate carboxylase (PEPCase) and glutamate synthase NADH-dependent (NADH-GOGAT); fumarase and glucose-6P dehydrogenase (G6PDH) activities in the roots (**left**) and leaves (**right**) of barley plants grown under control conditions (black bars) and exposed to 1 mM Ni^2+^ (grey bars). Results are average of at least three to five different measurements ± standard error (error bars). Asterisks indicate p values (* *p* ≤ 0.05, ** *p* ≤ 0.001) in Ni^2+^-exposed plants with respect to control plants at the same time of sampling.

In the leaves, PEPCase activity was 27.7 ± 1.4 nmol min^−1^ mg^−1^ protein. A gradual increase was observed upon Ni^2+^ exposure, resulting in a final activity 83% higher than controls after 7d, up to 48.2 ± 0.43 nmol min^−1^ mg^−1^ protein (Figure 4, left).

In roots, NADH-GOGAT activity increased by 46% after 9 h up to 15.2 ± 0.5 nmol min^−1^ mg^−1^ protein upon Ni^2+^ exposure; then, activity gradually returned after 7 d to the similar, initial levels, to 11.8 ± 0.16 nmol min^−1^ mg^−1^ protein (Figure 4, left).

NADH-GOGAT rate gradually and continuously increased in leaves: doubling from 0.81 ± 0.013 nmol min^−1^ mg^−1^ protein to 1.58 ± 0.023 nmol min^−1^ mg^−1^ protein after 7 days of Ni^2+^ (Figure 4, right).

Total fumarase activity drastically increased five times in barley roots upon Ni^2+^ exposure, from 90 ± 5 nmol min^−1^ mg^−1^ protein to 456 ± 10.5 nmol min^−1^ mg^−1^ protein (Figure 4, left).

In the leaves, fumarase activity upon Ni^2+^ exposure slightly increased by 18% up to 150 ± 2.8 nmol min^−1^ mg^−1^ protein after 1d, then returned to its initial level, 130 ± 0.5 nmol min^−1^ mg^−1^ protein (Figure 4, right).

Total G6PDH activity was assayed both in roots and leaves of barley plants exposed to 1 mM Ni^2+^ at different times of exposure. G6PDH activity in untreated plants was 110 ± 1 nmol min^−1^ mg^−1^ protein in roots; a prompt and stable 35-fold increase in G6PDH activity was observed upon 1 mM exposure to Ni^2+^ after 1d (Figure 4, left). After 7d of exposure, Ni^2+^ exposure caused a decrease in root G6PDH activity to the initial levels.

In leaves, Ni^2+^ increased G6PDH activity 3-fold after 1d, from 289 ± 0.7 nmol min^−1^ mg^−1^ protein to 85 ± 1.5 nmol min^−1^ mg^−1^ protein (Figure 4, right).

### 2.5. Changes in Presence of Proteins and Enzymes upon Ni^2+^ Stress in Barley Leaves

#### 2.5.1. Proteins and Enzymes Involved in Photosynthesis and Carbon Metabolism

**Figure 5:**
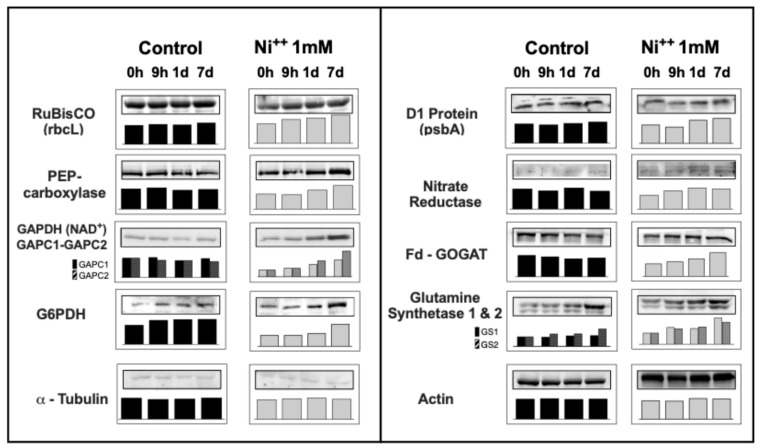
Immunoblotting of leaf extracts of plants grown under control conditions (left columns) and plants exposed to 1 mM Ni^2+^ (right columns) at given times, using antibodies directed vs. RuBisCO Large subunit (RbcL); phosphoenolpyruvate carboxylase (PEPCase); glyceraldehyde-3P dehydrogenase, NAD^+^ dependent (GAPC1, cytosolic; GAPC2, cytosolic); nitrate reductase; glutamine synthetase (GS1, cytosolic isoform; GS2, chloroplastic isoform); ferredoxin-dependent glutamate synthase (Fd-GOGAT). In the lower row, b-tubulin and actin immunoblotting are shown as control for equal loading. Densitometric analyses—as bar charts—for each immunoblotting are shown. Images are representative of at least three different immunoblotting results.

Protein D1, one of the main components of PSII, increased its presence with Ni^2+^ by 35% after 7d of Ni^2+^ exposure. Also, RuBisCO Large Subunit (RbcL) increased by 30% after 1d, and up to 45% after 7d of Ni^2+^ exposure (Figure 5). Immunoblotting analysis confirmed a similar increase in PEPC in the leaves (+55% after 7d—Figure 5).

Interestingly, GAPDH-NAD^+^ (cytosolic, glycolitic) proteins changed differently upon Ni^2+^ exposure: GAPC1 increased 3.7-fold after 7d, while GAPC2 rose 2.5-fold after 7d.

Glucose-6P dehydrogenase total protein increased quite regularly, by 20% after 1D, and its presence result doubled after 7d of Ni^2+^ exposure.

#### 2.5.2. Enzymes Involved in Inorganic Nitrogen Assimilation and Metabolism

Nitrate reductase protein rose by 50% after 1D, then remained stably increasing up to 7d upon Ni^2+^ exposure.

Fd GOGAT increased by 40% after 1d, and rose further up to +90% 7d after Ni^2+^.

Glutamine synthetase isoforms similarly increased upon Ni^2+^ exposure: GS1 (cytosolic) increased by 54% after 1d and up to 2-fold after 7d, while GS2 (chloroplastic) increased by 40% after 1d and up to 2.3-fold after 7d (Figure 5).

### 2.6. Changes in Gene Expression upon Ni^2+^ Stress in Barley Leaves

**Figure 6:**
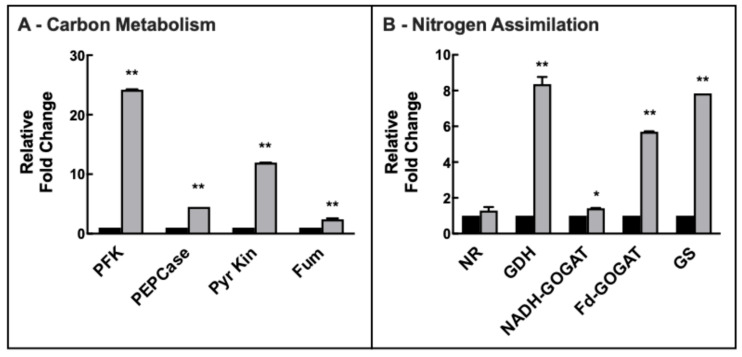
Changes in the expression of enzymes of carbon metabolism (**A**) and nitrogen assimilation (**B**) in leaves of barley plants grown under control conditions (black bars) and after 7 d (light grey bars) of 1 mM Ni^2+^ exposure. Variations are indicated as relative fold-change with respect to controls. Legend Panel A: PFK, phosphofructokinase (ATP dependent); PEPCase, phosphoenolpyruvate carboxylase; Pyr Kin, Pyruvate kinase; Fum, Fumarase. Legend Panel B: NR, Nitrate Reductase; GDH, glutamate dehydrogenase; NADH-GOGAT, glutamate synthase, NADH dependent; Fd-GOGAT, glutamate synthase, Fd dependent; GS, glutamine synthetase. Results are average of at least three different measurements ± standard error (error bars). Asterisks indicate p values (* *p* ≤ 0.05, ** *p* ≤ 0.001) in Ni^2+^-exposed plants with respect to controls at the same time of sampling.

The expression of phosphofructokinase (PFK) showed a strong increase in green tissues, over 24-fold after 7 d of Ni^2+^. This is in accord with the robust 12-fold increase in pyruvate kinase expression in leaves after 7 d of Ni^2+^. Interestingly, PEPCase expression increased 4.5-fold, and fumarase expression showed a 2.4-fold raise upon Ni^2+^ exposure in the leaves (Figure 6A).

Nitrate reductase expression remained substantially unchanged (+30% expression), similarly to NADH-GOGAT (+40%) upon Ni^2+^ exposure. More strong increases were observed in the expression of glutamine synthetase (+7.8-fold), Fd-GOGAT (+5.7-fold), and, interestingly, glutamate dehydrogenase (+8.4-fold) upon Ni^2+^ exposure (Figure 6B).

## 3. Discussion

The abiotic stress response in plants has become increasingly important in recent decades, and research about the effects of heavy metal pollution on crops has been deeply developed [3]. A general description of the effects of Ni^2+^ on the main plant phenotypic parameters has been summarized [9,24].

Interestingly, investigation on the damage caused by Ni^2+^ accumulation in the environment remained elusive due to the complex effects of this pollutant on living organisms [26]. Furthermore, when these effects were described, the response of plants to Ni^2+^ stress has not been specifically defined, and most previous work generally indicated the activation of ROS-responding pathways to reduce the oxidative stress induced by Ni^2+^ [23].

Poaceae present a low sensibility to heavy metals [27], and particularly Ni^2+^: *Z. mays* showed lower uptake, bioaccumulation, and translocation factors upon Ni^2+^ exposure with respect to other species, thus resulting in a reduced phytotoxicity of Ni^2+^ in these plants [28].

Preliminary experiments on barley plants grown in hydroponics indicated that a level of 1 mM Ni^2+^ and an exposure up to 7 days could mimic the long-term effects of Ni^2+^ accumulation in soils. In this regard, it is worth noting that it is widely assessed that the effects of metals are dose/time dependent, as demonstrated by previous papers [19,24], even at higher levels of Ni^2+^ [29] and in the same barley variety grown in hydroponics by our research group using Cd^2+^ [30].

Moreover, a recent review on the effects of Ni^2+^ stress in plants clearly demonstrates that—for most plants, and particularly cereals—Ni^2+^ exposure at concentrations of about 350 μM (in the same order of magnitude of 1 mM Ni^2+^ utilized in our experiments) and shorter than 14d had negligible effects on protein content, especially in monocot grown in hydroponics. Furthermore, it has been reported that more evident damage was observed in those studies where plants were exposed for longer periods (14 d or more) [24].

The utilization of hydroponics—enhancing the pollution effect—and the well-known dose/time dependent effect of HMs [6,30] could be useful to simulate the effective Ni^2+^ stress in a crop such as barley [8]. Namely, this study is mainly focused on the expression, presence, and activity of enzymes of primary metabolism; thus, we chose higher concentrations (1 mM) and shorter durations (no more than 7 d) in order to more evidently show changes in gene expression, protein presence, and enzymatic activities.

### 3.1. Cell Damage and Growth

Heavy metal toxicity decreases root length and changes their morphology in *Brassica juncea* [31] and *Brassica oleracea* [32]. Reduced growth was reported in Ni^2+^-exposed maize [16] and wheat [33]. Similarly, we observed an evident inhibition of growth in barley roots due to their contact with the soil pollutants, and comparable effects were noted in leaves. As result, the physiological growth observed in control plants is severely reduced, if not halted, during 1 mM Ni^2+^ exposure.

Water Content (WC) and Relative Water Content (RWC) in the leaves were measured after 1 mM Ni^2+^ exposure: a slight increase in Water Content was observed in leaves (Supplemental Figure S1A). Relative Water Content did not change upon Ni exposure, suggesting that Ni^2+^-treated leaves have a lower DW, while leaf hydration was not apparently altered by Ni^2+^ (Supplemental Figure S1B).

Furthermore, both RAPD analyses and the COMET assay witnessed a present cell nuclei damage in barley leaves exposed to Ni^2+^. In this regard, it has previously been demonstrated that high levels of heavy metals induce genotoxic effects in plants: exposure to cadmium and aluminum caused DNA degradation in *Vicia faba* and *Allium cepa* [34]. Specifically, Ni^2+^-treated plants showed severe genotoxic damage revealed by RAPD profiles [35] and COMET assays [36], confirming these techniques as valuable biomarker assays for the evaluation of genotoxic effects induced by Ni^2+^ in plants [36,37].

### 3.2. Photosynthetic Efficiency

Ni^2+^ stress harms the photosynthetic apparatus, consequently causing a decrease in photosynthetic efficiency. First, the response to the oxidative stress shifts reductants from the photosynthetic path towards ROS-responding pathways. Second, the diversion of this flow of electrons not only reduces carbon fixation but affects all those pathways which rely on the electron flow to sustain primary metabolism, such as nitrogen assimilation, sulfur compound synthesis, and general cell homeostasis mechanisms. As a consequence, the activation of those pathways—usually disconnected (or less active) during photosynthesis—is required to sustain the increased request of electrons diverted for the response to oxidative stress.

The analysis of the photochemical indexes confirmed that Ni^2+^ significantly reduced the functionality of the photosynthetic apparatus in barley leaves. Plants exposed to Ni^2+^ showed a 15% reduction in the maximum photochemical efficiency of PSII (F*v/*F*m*) and 33% quantum yield of ΦPSII after 7 d. The non-photochemical quenching (NPQ) remained initially unaffected, then continuously increased by 50% after 7 d.

Therefore, it is not unexpected that leaf cells increased the presence of both D1 protein (PsbA) and RuBisCO RbcL at a similar extent, 35–45%, suggesting a compensating effect due to the loss of photosynthetic efficiency [38].

### 3.3. Carbon Metabolism

In leaves, PEPCase increased activity (70%), presence (55%), and expression (2.5-fold) after Ni^2+^ exposure. Previously, a rise in PEPCase activity has been linked to increase in respiration induced by photosynthetic damage caused by Ni^2+^ [39].

Taken together, these results suggest an increase in photorespiration, indicated by the compensating effect of RuBisCO protein and evident rise in “dark” CO_2_ fixation by PEPCase. Furthermore, the increase in PEPCase suggests an increased flux of carbon in the TCA cycle. It is well known that HMs reduce photosynthetic carbon assimilation [40] and affect both dark CO_2_ fixation [41] and the TCA cycle [42].

Interestingly, fumarase activity (TCA cycle) increased 4-fold in the roots, suggesting a major response in mitochondria under HM stress in non-photosynthetic tissues. In barley, this can result in an increase in malic acid, citric acid, other organic intermediates [23], and aminoacids [24]. This rise in organic anions (malate and citrate) was also observed in *B. oleracea* under Zn^2+^ stress [32] and in wheat exposed to Ni^2+^ [39]. These organic acids are necessary to facilitate the transport of metals in the leaves (malate) and/or binding the metal for sequestration in the vacuoles (citrate), or to maintain metal ion homeostasis [42]. In the roots, high levels of organic acids could be induced by the need of dicarboxylate exudates for chelating heavy metals ions in the soil [43]. These results would suggest a complex regulation of the TCA cycle in heterotrophic tissues upon HM stress in higher plants [44] that should be further and better investigated in the future.

In the leaves, changes in PEPCase activity would suggest an involvement of the TCA cycle in photosynthetic tissues [44]. The increase in fumarase activity in leaves reinforces the hypothesis of a possible role of the TCA cycle during Ni^2+^ stress response. On the other hand, it is noteworthy that we previously observed an increase in PEPCase activity under Ni^2+^ stress, while, in wheat, we reported a decrease in PEPCase activity and occurrence upon drought [45].

This poses the question of the great variability of abiotic stress response in cereals, as observed also in the variability of Ni^2+^ stress response in rice varieties, underlining the need for further investigation on these complex mechanisms [18]. Our data confirm the improvement in glycolytic flux, with an evident increase in the expression of PFK, pyruvate kinase, and glyceraldehyde 3P dehydrogenase-NAD^+^, suggesting the activation of carbon oxidative pathways under metal stress.

The oxidative pentose phosphate pathway (OPPP), and namely the first and main regulating enzyme, G6PDH, showed a robust increase in activity and presence in barley leaves under Ni^2+^ stress, sustaining a main role of this pathway in the response both to N assimilation [46] and abiotic stress in higher plants [47,48].

In this context, keeping in mind that OPPP presents a highly complex regulation [49], and knowing that distinct and differently regulated G6PDH isoforms occur in plant tissues [47], the changes observed require further and deeper investigation in the near future.

### 3.4. Nitrogen Assimilation

Nitrogen assimilation is affected by Ni^2+^ stress, modifying the rates of several enzymatic activities [16].

Our data show that Ni^2+^ exposure induced evident increase in most of the enzymes involved in primary nitrogen assimilation: nitrate reductase, GS1, GS2, Fd-GOGAT, and NADH-GOGAT [50]. These results confirm those previously obtained on tomato roots, where both the activities and expression of inorganic nitrogen transporters and nitrogen assimilating enzymes were clearly enhanced by Ni^2+^ exposure [25]. Therefore, in Ni^2+^-exposed plants, the increased nitrogen recycling of leaf ammonium is confirmed by the augmented photorespiration cycle and increased nitrate reduction and chloroplastic N assimilation.

In wheat seedlings exposed to 0.1 mM Ni^2+^, a diversion of nitrogen assimilation was observed from the NR/GS/Fd-GOGAT pathway towards NADH-GOGAT/GDH and transaminases activation in leaves [33]. In rice, Ni^2+^ exposure up to 0.2 mM induced a limitation in inorganic N uptake, a decrease in the activities of nitrogen assimilating enzymes such as NR, GS, and GOGAT, and an increase in glutamate dehydrogenase (GDH) activity [19]. On the other hand, Zn limitation caused a sensible increase in GS activity in both lattuce and *B. olereacea* cv. Bronco [51].

In barley leaves, upon Ni^2+^ exposure, the presence of NR, GS1, GS2, and Fd-GOGAT continuously increased. Similarly, an increase in NADH-GOGAT activity was observed. Furthermore, the increase in GDH and NADH-GOGAT activities has been demonstrated as a general constitutive response in cereals upon abiotic stress [45].

These different behaviors can be ascribed to different responses of different cereal species and varieties under metals—namely Ni^2+^ [18].

The robust increase in the expression of Fd-GOGAT and GS2 suggests an increased recycling of inorganic nitrogen in the leaves [52]. On the other hand, the evident increase in GDH expression reinforced the role of this enzyme in Ni^2+^ stress response.

Alternative N assimilation through GDH under Ni^2+^ stress could be required to reduce ammonium toxicity, synthesizing glutamate for the production of stress-responsive metabolites such as gluthatione [53].

Previous studies have shown an association between OPPP, nitrogen assimilation, and the stress response, due to an increase in activity and levels of G6PDH during the exposure of plants to various nutritional and abiotic stresses [54,55,56].

Our data show an evident increase in total enzymatic G6PDH activity in the leaves of plants exposed to 1 mM Ni^2+^. Remarkably, a close correlation between variation in G6PDH and activities of GOGAT has been demonstrated, to provide the reductants necessary for GOGAT activity. Many studies confirm the close association between G6PDH and nitrogen assimilation in higher plants metabolism ([47,49] and references therein), and therefore these results suggest a specific role of the oxidative pentose phosphate pathway to contrast the effects of Ni^2+^ stress in barley.

## 4. Materials and Methods

### 4.1. Plant Material and Growth Conditions

Barley (*Hordeum vulgare* cv. Nure) seeds provided by the CRA-CPG “Centro di ricerca per la genomica e la postgenomica animale e vegetale (GPG)” (Fiorenzuola d’Arda—PC—(Fiorenzuola d’Arda, PC, Italy), courtesy of Dr. Cattivelli. *Hordeum vulgare* Nure is a barley distic variety cultivated in southern Italy. This variety presents a good drought resilience, and it is generally used for forage and brewing.

Seeds of barley were germinated on moistened paper in the dark for 5–7 days at 25 °C. The seedlings were transferred to hydroponic culture (Hoagland solution) according to [54,57], in 500 mL dark bottles (50 plants/bottle). Briefly, plants were grown for 7 days on a nutrient solution at pH 6.5 [50 mM KH_3_PO_4_, 50 mM K_2_HPO_4_, 1 mM CaCl_2_, 1.25 mM K_2_SO_4_, 1 mM MgCl_2_, 10 mM KNO_3_, 12.6 μM FeSO_4_—EDTA, 13 μM H_3_BO_3_, 0.24 μM CuSO_4_, 0.35 μM MnSO_4_, 1.5 μM ZnSO_4_, 0.008 μM (NH_4_)_6_Mo_7_O_24_] in a controlled cabinet at 20 °C under light intensity of approximately 180 μmol photons m^−2^ s^−1^ in a 16 h light/8 h dark regime, relative humidity 50–60%. Hydroponics were continuously bubbled with air using an aquarium air pump; the nutrient solutions were controlled for pH and adjusted daily.

All the reagents for the culture medium were from Carlo Erba (Milan, Italy) and Sigma-Aldrich-Fluka (Milan, Italy). At the seventh day, seedlings were randomly separated into controls and Ni^2+^-treated plants. The latter plants were exposed to 1 mM NiCl_2_.

Samples were collected at given times after Ni^2+^ exposure, or under control (no Ni^2+^) conditions, from two separated vessels for each treatment. Preliminary experiments using 0.5 to 2 mM NiCl_2_ were designed to analyze Ni^2+^ visible effects on barley plants, general growth, enzyme activities, and expression.

### 4.2. Growth Variation in Barley Plants

Changes in growth of barley plants exposed to Ni^2+^ were measured at given times (9 h, 1 d, 7 d) on five to ten randomly chosen plants. Controls were represented by 0 h time (start of Ni^2+^ exposure) and in parallel control plants grown in the same conditions unexposed to metal.

### 4.3. Photosynthesis Measurements

Fluorescence measurements were carried out by a portable FluorPen FP100max fluorimeter equipped with a light sensor (Photon System Instruments, Brno, Czech Republic). The ground fluorescence signal, F0, was induced on 30′ dark adapted leaves using an internal light of about 1–2 μmol m^−2^ s^−1^. The maximal fluorescence level in the dark, F*m*, was induced by a 1s saturating light pulse of 3000 μmol m^−2^ s^−1^. The maximal PSII photochemical efficiency, F*v*/F*m*, was calculated as (F*m* − F0)/F*m*. For the fluorescence measurements in the light, the fluorimeter (FluorPen FP100max) was equipped with an open leaf-clip suitable for measurements under ambient light. The quantum yield of PSII electron transport (ΦPSII) and non-photochemical quenching (NPQ) were measured and calculated as in [58]. Five fully expanded leaves for each treatment were chosen for the measurements.

### 4.4. Comet Assays

Comet assays were performed on leaves and roots of barley plants to determine possible DNA strand breaks.

About 150 mg of tissue was cut with a sharp blade in 1.5 mL of Tris 400 mM, pH 7.5, for 15 min to allow the protoplast release. The cellular suspension was then filtered with 20 μm Miracloth and, at 500 μL suspension, was added to 500 μL LMA (1% low melting agarose in Dulbecco’s PBS). On a microscope slide covered by 1% NMA (normal melting agarose), 80 μL of the mix (suspension + LMA) was added and then stored for 5 min at 4 °C.

Cells were lysed for 1h at 4 °C using a specific buffer (2.5 mM NaCl, 100 mM EDTA, 10 mM Tris, 1% Triton X100, 10% DMSO, pH 10.0).

Microscope slides were washed two times in 400 mM Tris-HCl pH 7.5 and then put for 15 min in the electrophoretic buffer (Na_2_EDTA 1mM, NaOH 300 mM, pH > 13) to allow DNA release. After 15 min, an electrophoresis was made on these samples, 25 min at 25 V, 300 mA. Slides were removed from the electrophoresis chamber, rinsed, and neutralized in 400 mM Tris-HCl, pH 7.5. DNA was visualized by fluorescence microscopy (Nikon Eclipse E1000, Nikon Europe B.V., Amstelveen, The Netherlands) after staining with a 50 μg DNA-binding dye (DAPI, AppliChem) for 3 h. Comets observed were analyzed by ImageJ v.1.51 and Casplab software.

### 4.5. Preparation of Protein Extracts

Extracts for determination of enzymatic activities were prepared from 0.3 g of leaves or roots. Samples were mechanically homogenized (TissueLyser, QIAGEN S.r.l., Milan, Italy) at 50 Hz for 2 min (five times) using stainless steel beads (5 mm) under ice-cold conditions in 600 μL of extraction buffer. Homogenates were centrifuged at 13,000 rpm (20,000× *g*—Eppendorf© centrifuge 5415D, Eppendorf srl, Milan, Italy) for 20 min at 4 °C. The supernatant fraction was transferred to fresh eppendorf© tubes (1.5 mL) and clarified by centrifuging for 10 min at 4 °C. The pellet was discarded, and the clear supernatant was utilized in assay activities.

Protein concentrations were determined using the Coomassie blue method (Bio-Rad 500-0006, Bio-Rad Italia, Segrate (MI), Italy); bovine serum albumin was utilized as the standard. Chemicals for protein and enzymatic analyses were all analytical grade and purchased from Sigma-Aldrich Chemical Co., Ltd., St. Louis, MO, USA, unless otherwise noted.

### 4.6. Extraction and Assays of Enzymes

Enzymatic activities and protein concentrations were measured using a Cary 60 spectrophotometer (Agilent Technologies, Santa Clara, CA, USA) equipped with an 18 cell holder and connected to a personal computer equipped with Cary WinUV software.

Phosphoenolpyruvate carboxylase. PEPCase activity was measured in a coupled assay with malic dehydrogenase as in [50]. The NADH extinction coefficient was 6.22 mM^−1^ cm^−1^ at 340 nm, and PEPCase specific activity is expressed as nmol NADH oxidized mg^−1^ protein · min^−1^.

Glucose-6P dehydrogenase. G6PDH enzymatic activity was determined following NADPH formation [59]. Specific activity is expressed as nmol NADP^+^ reduced mg^−1^ protein · min^−1^.

Glutamate synthase NADH-dependent. NADH-GOGAT activity was assayed as in [45] by monitoring NADH oxidation at 340 nm. The NADH extinction coefficient was 6.22 mM^−1^ cm^−1^ at 340 nm. NADH-GOGAT activity is expressed as nmol NADH oxidized mg^−1^ protein · min^−1^.

Fumarase. Fum activity was measured by monitoring the conversion of L-malate in fumarate. A reaction mixture of L-malic acid was prepared by utilizing 7 mg · mL^−1^ of L-malic acid in 100 mM K-phosphate buffer (pH 7.6). Changes in absorbance at 240 nm were measured and converted as malate using an extinction coefficient of 2.24 mM^−1^ cm^−1^. Fumarase activity is expressed as nmol malate oxidized mg^−1^ protein · min^−1^.

### 4.7. Gel Electrophoresis (SDS-PAGE) and Immunoblotting Analyses

Reagents and equipment for SDS-PAGE and immunoblotting are from Bio-Rad (Milan, Italy) unless otherwise noted.

SDS-PAGE and immunoblotting analyses were performed as previously described [60]. Details are provided in the Supplemental Materials.

The separated polypeptides were transferred to a PVDF membrane and incubated with antibodies (from Agrisera, Vännäs, Sweden—unless otherwise noted) vs. RuBisCO large subunit (RbcL—EC 4.1.1.39, AS03 037); D1 protein (PsbA—AS05 084); glutamine synthetase 1 & 2 (EC 6.3.1.2, AS08295); glyceraldehyde-3-P dehydrogenase (GAPDH NAD+—GAPC1 and GAPC2–EC:1.2.1.12, AS15 2894); assimilatory nitrate reductase (EC 1.7.1.1, AS08310); glucose 6P dehydrogenase (G6PDH—EC 1.1.1.49; Merck Sigma Aldrich A9521); barley Fd-GOGAT (EC 1.4.7.1) (gentle gift of Prof. A. Marquez, Sevilla, Spain); and Amaranthus edulis PEPCase (EC 4.1.1.31) (gentle gift of Prof. R.P. Walker, Sheffield, UK).

Immunoblottings using anti-actin (AS13 2640) and anti-Tubulin β-chain (AS10 681) (Agrisera, Vännäs, Sweden) were used as reference for equal loading of the lanes. Bands were visualized by enhanced chemiluminescence and visualized using Chemidoc XRS (Bio-Rad).

### 4.8. RNA Isolation, cDNA Production and Real-Time PCR Analyses

Total RNA was extracted from 300 mg · FW leaf material using Trizol reagent. cDNA was prepared using a ready to use kit (PrimeScript; Takara Bio, Shira, Japan).

Gene expression analysis was carried out by qRT-PCR using an ABI 7900 HT (Applied Biosystems, Foster City, CA, USA) and Platinum SYBR Green qPCR SuperMix (Life Technologies, Carlsbad, CA, USA). Leaf samples of plants grown in control conditions were used as calibrators. α-Tubulin-2 (Y08490.1) served as endogenous reference gene.

Primers for each gene were designed as in Supplemental Table S1. Quantitation of gene expression was carried out using the 2(-Delta Delta C(T)) as in [61].

### 4.9. Statistical Analysis

Each experiment or measurement was repeated three times and made in three to five replicates. Values were expressed as mean ± standard deviation (SD). The statistical significance between Ni^2+^ stressed and control plants was evaluated through Student’s *t*-test (* *p* < 0.05, ** *p* < 0.001; (a) *p* < 0.05, (b) *p* < 0.001).

Immunoblotting images and densitometric analyses were obtained using Image Lab software (Bio-Rad) and were representative of at least three independent measurements.

Photosynthesis data were assessed by one-way ANOVA followed by the Student Newman Keuls test for multiple comparisons. Differences were considered statistically significant at *p* < 0.05. The processing of the data was performed using statistical functions available in Sigma-Plot 12.0 software (Jandel Scientific, San Rafael, CA, USA).

## 5. Conclusions

Barley plants exposed to Ni^2+^ show a complex response. In the roots, a primary burst of mitochondrial respiratory processes sustains the stress response.

In the leaves, a decrease in the photosynthetic efficiency parameters was observed, while RuBisCO occurrence remained unchanged. Increased PEPCase activity and expression were observed, and data suggest an improved photorespiratory recycling of ammonia.

We suggest that, under Ni^2+^ stress, leaf cells are able to flank the main GS/GOGAT cycle with N assimilation via the GDH reaction, possibly to save ATP moieties utilized to counteract Ni^2+^ toxicity, e.g., increasing plasmalemma/vacuolar metal pump activities.

This diversion requires an increased production of carbon skeletons through glycolytic flux, and an increased synthesis of both dicarboxylates derived from the TCA cycle and related aminoacids.

Using this trick, photosynthetic cells could save and divert energy, as we previously noted in specific barley landraces under salinity [62] and wheat landraces in different arid environments [63].

Furthermore, the data presented here also suggest the role of OPPP and, more specifically, G6PDH in sustaining basal cell metabolism under stress.

More appropriate and specific studies are required to better clarify some aspects of this complex set of responses to Ni^2+^ stress and disclose the roles of these enzymes in response to abiotic stress in plants.

## Data Availability

The original contributions presented in this study are included in the article/Supplementary Material. Further inquiries can be directed to the corresponding authors.

## Supplementary Materials

The following supporting information can be downloaded at https://www.mdpi.com/article/10.3390/plants14182927/s1. Supplemental Materials and Methods: Water Content and Relative Water Content analyses; RAPD analyses. Figure S1: Water Content and Relative Water Content in barley leaves. Figure S2: RAPD analyses. Table S1: List of oligonucleotide primers designed for qRT-PCR analyses. Table S2: Nickel content in barley tissues.

## Abbreviations

The following abbreviations are used in this manuscript:

Fd-GOGAT	Ferredoxin-dependent glutamine oxoglutarate aminotransferase (glutamate synthase)—EC 1.4.7.1
G6PDH	Glucose-6P dehydrogenase—EC 1.1.1.49
GS1/GS2	Glutamine synthetase 1(cytosolic)/glutamine synthetase 2 (chloroplast)—EC 6.3.1.2
NADH- GOGAT	NADH-dependent glutamine oxoglutarate aminotransferase (glutamate synthase)—EC 1.4.1.14
NR	Nitrate reductase—EC 1.7.1.1
PEPCase	Phosphoenolpyruvate carboxylase—EC 4.1.1.31
PMSF	Phenyl-methyl-sulfonyl-fluoride
RuBisCO	Ribulose-1,5-bisphosphate carboxylase/oxygenase—EC 4.1.1.39
