# Supplementary material for: High Dose of Nickel Unbalances Carbon Metabolism and Nitrogen Assimilation in Barley (Hordeum vulgare L.)
Source: Plants (Basel). 2025 Sep 20;14(18):2927. doi: 10.3390/plants14182927 (PMC12473449; doi:10.3390/plants14182927)
Supplement: Supplementary file 1 [file plants-14-02927-s001.zip › plants-3851526-supplementary.pdf]

Plants - MDPI

High dose of Nickel unbalances carbon metabolism and nitrogen assimilation in barley (*Hordeum vulgare* L.)

Alessia De Lillo, Ivana De Rosa, Giorgia Capasso, Giorgia Santini, Concetta Di Napoli, Noemi Russo, Ermenegilda Vitale, Stefania Grillo, Sergio Esposito, Simone Landi

Supplemental Data

Supplemental Table S1 – List of oligonucleotide primers designed for qRT-PCR analyses

Genes	Sequence
PFK Forward	GGGCCTGGTTGATCTTTTCAC
PFK Reverse	CAAGAAACGCCACCCATACC
PEPcase – Forward	ACAATGTACTGGATTGGGCG
PEPcase – Reverse	AGCTAGAAGGCACCACAAAAT
Pyruvate kinase Forward	TATGGAGGTGAAGGAGGATGAC
Pyruvate kinase Reverse	GTTTTCAGACTCTACACGGCG
Fumarase – Forward	AGCCTCAGAGTTTTGACGGA
Fumarase – Reverse	TGGTAACCTGAACGCTTGTGT
Nitrate reductase Forward	CCTTAGCCTTGAGATAAGGGG
Nitrate reductase Reverse	CAAGACAGACCAAGAACTCCCT
GDH Forward	TCGTGTAATCCGCAGCTGAA
GDH Reverse	CAGGCTCACGCGTCATCAA
NADH-GOGAT Forward	CGCCGTAGGATCAGACAAGT
NADH-GOGAT Reverse	CTCCCCGTTTGTTCCACTGA
Fd-GOGAT Forward	GGGGGTCTTCCTCTAACGAT
Fd-GOGAT Reverse	AGCTTTCCCGCTCTCTTCTC
GS Forward	AACTACGACGGATCGAGCAC
GS Reverse	CTCCTCGGAATGGGTCCTTG
Alfatub - Forward	CTCCATGATGGCCAAGTGTGA
Alfatub - Reverse	ATGTCGCTTGGTCTTGATGGT

Supplemental Table S2 – Nickel content in barley tissues.

Nickel content (µg Ni · g-1 DW)	roots	roots	leaves	leaves
	**0d**	**7d**	**0d**	**7d**
**Control**	10 ± 0.3	11 ± 2	3 ± 0.27	3 ± 1
**1 mM Ni^{2+}**		5270 ± 3		130 ± 30

Supplemental Materials and Methods

Determination of water content

Water Content (WC) and Relative Water Content (RWC), were measured on 5 barley leaves. Samples were weighted to determine Fresh Weight (FW); then leaves were hydrated for 4 hours by floating on deionized water and weighed to determine the turgid weight (TW); then samples were dried overnight at 70°C for dry weight (DW) determination.
The plant WC was derived from the formula:
WC = (FW-DW)/FW %
Relative Water Content (RWC), representing the water content of a given amount of leaf relative to its fully hydrated or fully turgid state, was calculated as follows:
RWC = (FW-DW)/(TW-DW) %.

RAPD analyses

Total DNA was extracted from 0.4 g of leaves by Isolate II Plant DNA kit (BioLine). DNA concentration in the samples was determined at 260 nm, and integrity was checked on 1.5% agarose gels. Leaves from barley plants were analyzed for RAPD markers using different primers supplied by Operon Technologies Inc. (Alameda, Calif., USA).
RT-PCR for DNA amplification was performed in a volume of 25 μL, containing the reaction buffer: 10 mM Tris-HCl pH 8.0, 50 mM KCl, 1.5 mM $MgCl_2$ plus 200 μM of each dNTP, 0.8 μM 10-base primer, 25 ng of template DNA, and 2 units of Taq DNA polymerase. DNA amplifications were performed using a thermal cycler (Bio-RAD T100). The cycle used involved one preliminary step of denaturation, followed by 40 cycles of denaturation – annealing – elongation.
PCR products were separated on 1.8% agarose gels by electrophoresis run in 1X TBE buffer (89 mM Tris-borate and 0.2 mM EDTA) and visualized with DNA loading buffer red (Bioline).
RAPD procedure was checked by repeating the analyses of samples 5 times, only bands showing consistent amplification were analyzed.

Ni^{2+} determination

Ni^{2+} content were determined on dried samples, previously grounded and digested in a micro-wave digestion unit (Milestone mls 1200 - Microwave Laboratory Systems). Ni^{2+} levels in samples were measured by atomic absorption spectrometry (Atomic Absorption Spectrometer SpectrAA 20 - Varian).

Maisto, G.; De Nicola, F.; Alfani, A. Long-term dynamics of soil metal concentrations at the urban area of Naples (Southern Italy) *Fres. Environ. Bull.* **2010,** *19,* 1762 - 1767

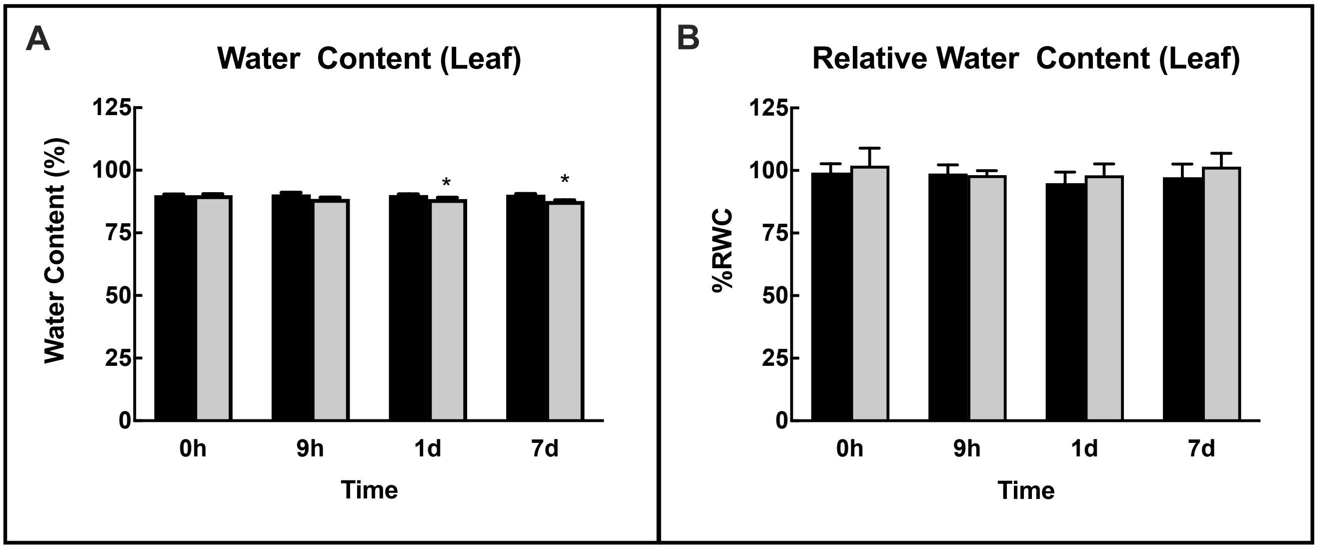

Supplemental Figure S1 – A, Variation in barley leaves of water content in control (black bars), and 1 mM Ni++-treated (light grey bars) at different times of exposure. B,, Variation in barley leaves of relative water (RWC) content in control (black bars), and 1 mM Ni^{2+}-treated (light grey bars) at different times of exposure.

Results are average of five to ten different measurements ± standard error (error bars). Asterisks indicate significant differences at $p \leq 0.05$ (*), in Ni^{2+}-treated plants with respect to controls at the same time of sampling.

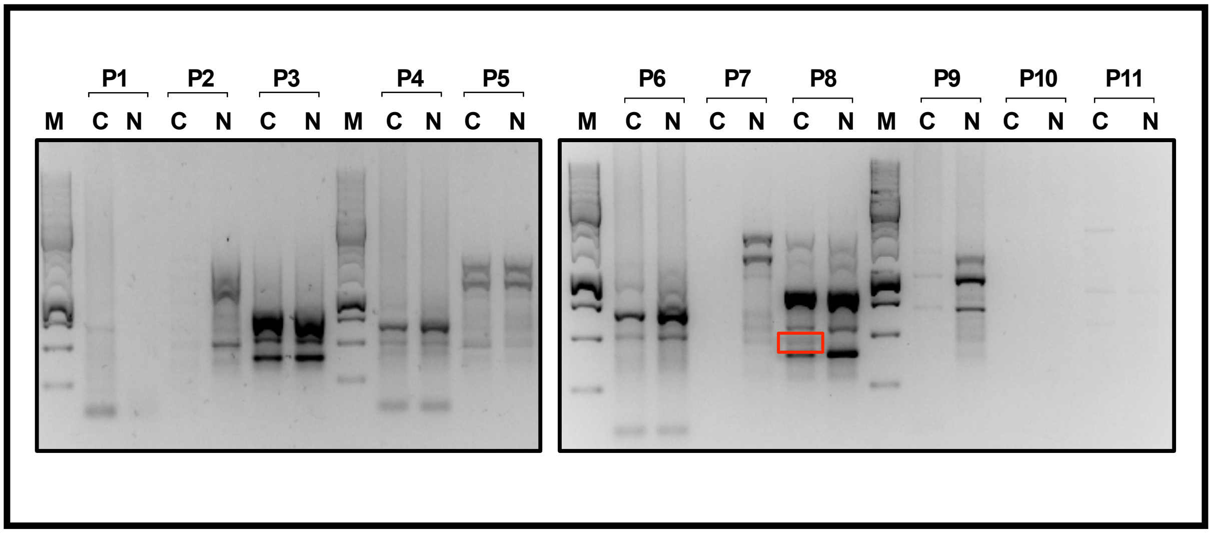

Supplemental Figure S2 - RAPD analyses of leaves of barley plants grown under control conditions (C) and 1d upon 1 mM Ni^{2+} exposure (N). The red square indicates the presence of different amplification pattern in one of the primers tested. Legend: P1 to P11 indicate unspecific primers used for RAPD and supplied by Operon Technologies Inc. (Alameda, Calif., USA). M, markers.
